# The case for value as a common currency in decision-making and intersystem competition

**DOI:** 10.3389/fcogn.2026.1767189

**Published:** 2026-03-20

**Authors:** Chandra Sripada

**Affiliations:** 1 Department of Psychiatry, Weinberg Institute for Cognitive Science, University of Michigan, Ann Arbor, MI, United States; 2 Department of Philosophy, Weinberg Institute for Cognitive Science, University of Michigan, Ann Arbor, MI, United States

**Keywords:** common currency, decision, expected utility theory, heuristics, intersystem competition, neuroeconomics, policy gradients, rationality

## Abstract

Inspired by economic theory, theorists in neuroeconomics and related areas of cognitive science contend that value serves as a common currency for selection among options. Although support for the view has grown over three decades, critics have recently raised serious concerns. They argue that there are a number of pathways to action that bypass value; dissimilar psychological systems do not share a common value currency; and the view cannot accommodate well documented kinds of “irrationality,” such as context effects. This article offers a systematic response. Key refinements are made to the common currency view, distinguishing common currencies that operate at two phases (decision-phase and learning-phase) and at two levels (options-level and meta-level). A further distinction is made between mechanistic common currency models utilized in cognitive science and axiomatic common currency models associated with expected utility theory and utilized in economics. These refinements help address the criticisms and yield a more nuanced understanding of the role of value in human decision-making.

## 1 Introduction

What explains why people do the things they do? Many influential answers have been given (Berridge, 2004). Behaviorists proposed learned associations (Skinner, 1963); ethologists appealed to intrapsychic hydraulic forces (Hinde, 1956); religious thinkers invoked an immaterial self that is the ultimate chooser.

Contemporary computational cognitive science, especially the field of neuroeconomics, offers a distinctive answer inspired by economic theory: People make decisions about what to do based on information about the expected value of their options, and they choose the option with highest value (Montague and Berns, 2002; Glimcher and Rustichini, 2004; Sanfey et al., 2006; Glimcher and Fehr, 2013; Serra, 2021). Value is a scalar variable that represents the subjective goodness or badness of an option, similar in some ways to the role played by utility in economic theories (though key differences are discussed below).

Critically, value also plays the role of a “common currency” for comparing options (Levy and Glimcher, 2011, 2012; Sugrue et al., 2005). People make decisions that trade off very different kinds of goods, for example food, money, safety, leisure, and social gains, among other things. Theorists claim that value serves as a common medium of exchange that allows for selection among dissimilar alternatives.

Over the last three decades, this “common currency of value” view has gained substantial empirical support, and the evidence base is broad and convergent. It spans multiple fields of cognitive science that use diverse methods, including behavioral, chronometric, computational, and imaging methods, and this evidence is reviewed in §3.

### 1.1 Criticisms of the common currency view

Despite these successes, the common currency view has come in for a number of lines of criticism. These arguments are scattered about the literature (Walasek and Brown, 2023; Bennett et al., 2021; Vlaev, 2018; Vlaev et al., 2011; Spurrett, 2009; Lichtenstein and Slovic, 2006; Slovic, 1995), but an important recent article by Hayden and Niv (2021) helpfully assembles several of the most important critiques.

One argument looks closely at algorithms from the reinforcement learning (RL) field (Sutton and Barto, 1998; Kaelbling, 1996). These algorithms are highly important for the common currency view because they provide computationally feasible and biologically plausible accounts to explain how agents can, using ongoing feedback from the environment, acquire information about the value of their options. There are, however, certain algorithms in the RL framework that are said to work by calculating the best actions “directly,” without the need for value estimation, for example so-called policy gradient algorithms (Sutton et al., 1999), and there is evidence for the operation of these algorithms in the human brain (Bennett et al., 2021). These “value-free” algorithms, it is argued, demonstrate that there are alternative pathways for decision and action selection that bypass calculation of value, seemingly at odds with the common currency view (Bennett et al., 2021; Hayden and Niv, 2021).

Another argument starts with the observation that the human brain is outfitted with numerous mechanisms and systems that contribute to choice and action guidance, many of which operate using distinctive computational principles (Sanfey and Chang, 2008; van der Meer et al., 2012) For example, some systems rely on a model of the world, other systems use “model-free” methods, still others use Pavlovian principles, and so forth. These observations raise doubts about whether there is a common medium of value that is shared across all these disparate systems allowing for commensuration across them

A third argument says that representing the value of one's options, while it certainly happens in some cases, is in fact relatively rare because it is much too computationally demanding. Instead, people use *heuristics* to rapidly select among a set of options without having to exhaustively calculate the value of the options (Tversky and Kahneman, 1974; Gigerenzer and Todd, 1999). It is now well established that heuristics are employed ubiquitously in human decision-making, and this fact is said to challenge the common currency view (Hayden and Niv, 2021).

A fourth argument says the common currency view, inspired by economic theory, has trouble accounting for certain forms of “irrationality.” People display a number of well-documented context effects—their preferences change depending on seemingly irrelevant features of the context in which they are elicited. Critics argue that the common currency view cannot account for these phenomena (Walasek and Brown, 2023; Vlaev et al., 2011; Slovic, 1995).

### 1.2 Defending the common currency view

The aim of this article is to systematically defend the common currency view against these criticisms while acknowledging that the critics' arguments involve key insights and yield important lessons. Three distinctions play key roles throughout the article.

The first distinction is between two phases at which a common currency can operate. Most theorists have assumed that a common currency of value will be implemented at the *decision-phase*, i.e., at the time that an agent confronts a decision among options. But there is an alternative approach in which a common currency is utilized during the *learning phase* to learn optimal, value-maximizing actions, and the agent does not necessarily need to access the common currency at the time of decision.

The second distinction is between two levels at which a common currency of value can operate. A common currency can be used to adjudicate among dissimilar options (“options-level currency”) or among competing systems, processes, or strategies (“meta-level currency”). It is argued that in key cases in which critics claim that a common currency is absent, a common currency is in fact operative at the meta-level.

The third distinction is between a *formal* and a *mechanistic* common currency. Standard expected utility theory from economics uses value as part of an axiomatic mathematical structure that reflects an agent's patterns of preferences over options. In neuroeconomics and related areas of cognitive science, in contrast, value is a mentally represented dimension that is used as a mechanism to quantify and compare the goodness or badness of the options, and where options stand on this dimension causally influences choice. As will be argued, this distinction between axiomatic and mechanistic common currency models is critical for accommodating and explaining “irrational” choice patterns.

The remainder of the article is divided into seven parts. Parts 1 and 2 clarify the common currency view and summarize some initial evidence from neuroeconomics and other areas of cognitive science in its favor. The next four parts take up four major arguments against the common currency view put forward by critics. It is argued that when key clarifications are made and certain misconceptions are addressed, the observations made by critics of the common currency view are seen to no longer count against it. Indeed, in many cases, these observations end up providing additional support for the common currency model. The concluding section reviews the overall state of the evidence for the common currency view.

## 2 What is a common currency of value?

There are a number of different ways that an agent can select its actions. For example, an agent can follow condition-action rules: if a certain situation is detected, a specific action is generated (Russell and Norvig, 2020), and Pavlovian responses are sometimes understood in this way, e.g., (Atkinson et al., 1987). Another approach utilizes “habit” mechanisms that, among the range of alternative actions available, select an action in proportion to the frequency with which that action has been produced before (Miller et al., 2019). Another approach relies on ordinal “rank” representations that encode which option is better but not how much better it is, and this approach is utilized in the influential decision-by-sampling model (Stewart et al., 2006; Noguchi and Stewart, 2018).

The common currency view says that selection among competing alternatives is explained in terms of an underlying common currency of value. A common currency is a single continuous dimension that represents the overall expected goodness or badness of the alternatives. Potentially dissimilar alternatives are located on this dimension, enabling comparison on a common scale. It is called a currency rather than a mere ordering because the dimension allows fine-grained magnitude information to be represented. In particular, to count as a common currency of value, the dimension must enable representation of the magnitudes of the values of alternatives, or magnitudes of differences among the values of alternatives, on a single shared scale.

Importantly, in a common currency model, the valuation process, the process by which values are assigned to options, can take different forms. For example, valuation can be multidimensional and hierarchical: the individual attributes of the options may first be assigned attribute-level values, which are then aggregated yielding an assignment of overall scalar values to the options (Stewart, 1992; Raiffa, 2006; Busemeyer et al., 2019; Roijers et al., 2013). Additionally, the model allows that valuation can be nonlinear in relation to the objective quantities of the underlying goods. This feature allows the model to capture phenomena such as diminishing marginal value (more of a good tends to be less valuable the more of it one already has; Caplin and Glimcher, 2014) and optimal set-points (a certain level of a variable, such as hydration, is valued most, and deviations in either direction are valued less; Keramati and Gutkin, 2014; Juechems and Summerfield, 2019; Dulberg et al., 2023).

Another clarification concerns the relationship between value as a common currency and the subjective experience of pleasure and pain. This article follows the perspective of most work in the neuroeconomic tradition that assumes that value as a common currency should be understood fundamentally in terms of its *decisional* effects—the core functional role of value is to serve as a basis for selecting among competing alternatives. It is likely that, in addition, representations of scalar value are richly interconnected with the subjective phenomenal experience of pleasure and pain. But these links are highly complex and potentially context-specific (Berridge and Kringelbach, 2008, 2015; Shenhav, 2024; Carruthers, 2024), and they are not explored here.

What is the scope of the common currency of value model? One approach is to claim the model applies to essentially all instances in which humans select among alternatives. A more modest approach says that the model applies very broadly across a number of psychological domains, but there may be important exceptions, and this is the approach is adopted for the purposes of this article. This more modest approach sees the debate about the common currency view as a series of potentially separable debates about how the model applies across individual psychological areas and domains. The sections that follow can be understood as defending the application of the common currency model in a number of areas in which critics have specifically claimed it does not apply.

A final clarification: The claim that people use a common currency of value as a basis for decisions is not put forward as a conceptual claim. It is a substantive, empirical claim about the type of mechanisms involved in explaining how options are selected, and the claim could be false. In particular, if in some domain, there is strong, convergent evidence that the mechanisms for selecting among options rely on condition-action rules or ordinal representations or some method other than a common currency of value, then the common currency view is thus shown to be false in that domain. The question of the overall state of the evidence for the common currency view and the falsifiability of the view is revisited in the final section.

## 3 An initial case for the common currency view

There are several lines of evidence that yield a compelling initial case for the common currency view.

One line of evidence comes from the reinforcement learning (RL) framework, which addresses the problem of how agents learn to behave in complex environments in ways that maximize achievement of their aims (Sutton and Barto, 1998; Kaelbling, 1996). There are a number of importantly different kinds of algorithms within the RL framework. The focus for now is on “value-based” algorithms, such as the temporal difference reinforcement learning algorithm (Sutton and Barto, 1998; Schultz et al., 1997; so-called “value-free” RL algorithms are discussed in the following section).

In the RL framework, an agent has a prespecified reward function, which identifies the things that count as “intrinsic aims” for the agent (Singh et al., 2009, 2010). These might include such things as nourishment, safety, and so forth, and each is associated with a scalar weight that quantifies its degree of importance to the agent. In value-based RL algorithms, the agent next uses feedback from the environment to learn the expected values of its actions in terms of cumulative future reward attainment. These expected value representations sum over potentially dissimilar reward types (i.e., dissimilar intrinsic goals such as nourishment and safety), thus allowing comparison of options in terms of a common currency of value.

There is substantial evidence that value-based RL algorithms do in fact play wide-ranging roles across multiple areas of cognition, including attention allocation (Theeuwes et al., 2022; Anderson, 2019; Failing and Theeuwes, 2018; Anderson and Kim, 2018; Callaway et al., 2021), action selection (Dolan and Dayan, 2013), working memory (D'Ardenne et al., 2012; Hazy et al., 2006), and executive control (Shenhav et al., 2013, 2016), among many other domains (see Sripada, 2025 for a review). These observations, in turn, provide evidence for the common currency view. This is because the view says that agents use a common currency of value to select the option with highest value. Value-based RL algorithms provide one important explanation, though certainly not the only one, for where information about the value of options ultimately comes from. Critically, it is unlikely that any future RL algorithm could completely do away with a notion of common currency of value, and recent work suggests alternative value-free approaches have trouble capturing key empirical datapoints (Hemmatian et al., 2024).

Another line of evidence for the common currency view comes from neuroimaging studies in which people are presented with goods of various kinds and their brain activation patterns are quantified (Glimcher and Rustichini, 2004; Serra, 2021; Montague et al., 2006). These studies have identified several neural regions, most prominently ventral striatum and ventral medial prefrontal cortex, that exhibit a trio of features. First, their activation scales with the expected value of the presented goods such that larger expected values, for example, larger expected monetary gains, are associated with stronger activation (Breiter et al., 2001; Knutson et al., 2005; Kable and Glimcher, 2007; Bartra et al., 2013). Second, these regions activate to a very broad range of goods, including food, attractive faces, monetary rewards, consumer items, social gains, and moral outcomes, among others (Bartra et al., 2013; FitzGerald et al., 2009; Kim et al., 2011; Lin et al., 2012). Third, activation in these regions is predictive of exchange rates or actual choice patterns among these goods (Levy and Glimcher, 2011; Plassmann et al., 2007; Smith et al., 2010). These observations have led some theorists to propose that these regions encode a common currency of value that enables choice over disparate option types (Levy and Glimcher, 2011, 2012; Kable and Glimcher, 2009).

Still another line of evidence for the common currency view comes from computational models of multiattribute decisions, such as decision field theory (Busemeyer and Townsend, 1993; Roe et al., 2001) and other sequential sampling-based models of preferential choice (Busemeyer et al., 2019; Trueblood et al., 2014; Howes et al., 2016), which have had substantial empirical success in capturing a range of behavioral phenomena, including patterns of choice and reaction time. These models are often applied to decision problems involving options with highly dissimilar attributes, for example vehicles that differ on cost, safety, and style. To enable comparisons across dissimilar dimensions, these models make a basic assumption that the agent can directly make pairwise comparisons of how much better an option is versus another option along a specific attribute dimension, i.e., the agent can make attribute-wise relative value assessments. Moreover, it is assumed value information arising from these comparisons can be additively summed up across comparisons involving different attributes. In short, then, these models make the assumption that value serves as a common scalar medium of comparison across dissimilar attributes and across options.

Finally, there is a venerable theoretical argument for the common currency model, which might be dubbed the “How else?” argument (see Berridge, 2004; Levy and Glimcher, 2011; Kable and Glimcher, 2009; McFarland and Sibly, 1975; Spurrett, 2021 for somewhat different versions of this general argument).

Value provides a highly efficient and parsimonious medium in which to encode countless tradeoffs across dissimilar goods, and to adjust these tradeoffs based on ongoing experience via learning algorithms (for example, RL algorithms). Without the use of value for this purpose, encoding tradeoffs between even just two goods becomes prohibitively complex (see Spurrett, 2021 for a detailed argument and Berridge, 2004 for a compelling illustration). Moreover, there is the further problem of how to adjust these tradeoffs if the goods change in decision-relevant ways—for example, if a certain food item no longer yields certain anticipated benefits. Based on these sorts of observations, theorists argue that value is a highly efficient solution to a ubiquitous problem, explaining why the use of value is widespread across psychological domains (Berridge, 2004; Levy and Glimcher, 2011; Kable and Glimcher, 2009; McFarland and Sibly, 1975; Spurrett, 2021).

These four lines of evidence make a strong initial case for the common currency view across a number of important domains of human psychology. Yet, a number of serious objections have been raised to the view, and these are discussed and responded to in the next four sections.

## 4 Policy gradients: Do some RL learning algorithms bypass value?

### 4.1 “Value-Free” RL algorithms

Many RL algorithms, such as the temporal difference algorithm, are value-based: They learn the expected value of the available options, enabling comparisons of these options on a common scale. Thus, they provide solid support for the common currency view.

But there are some RL algorithms that are said to be “value-free.” For example, so-called policy gradient methods are said to arrive at an action policy “directly” without calculating value (Sutton et al., 1999). Policy gradient algorithms, or closely related algorithms such as actor-critic methods (Joel et al., 2002), also have strong plausibility as algorithms implemented in the human brain, especially as a basis of routinized action control (Bennett et al., 2021; Joel et al., 2002). This fact potentially presents a serious problem for the common currency view: Policy gradient methods point to the presence of important pathways to action that bypass value.

However, as will be argued, a closer look at the workings of policy gradient algorithms calls this line of criticism into doubt.

In policy gradient algorithms, the agent assigns so-called action weights to its actional options for each environmental state, where these weights encode the probability of performing the associated action (the collection of action weights assigned across all states is called a “policy”). These action weights can initially be set randomly. The agent next observes rewards that arise from its actions as it navigates its environment (each full traversal of its environment is called an “episode”). The agent uses these observations from each episode to, via a gradient-based method, nudge the action weights up or down. That is, actions that were observed in an episode to yield higher subsequent cumulative rewards are nudged up and these actions are, going forward, performed with higher probability; actions observed to yield lower subsequent cumulative rewards are correspondingly nudged down. It can be shown that repeated application of this gradient-based process can converge on an optimal policy for action (Sutton et al., 1999).

Hayden and Niv (Hayden and Niv, 2021) argue that an agent selecting its actions through policy gradient methods does not choose their actions based on value. A policy gradient agent acts on the basis of action weights—these weights determine the probability of performing the respective actions. These weights, they argue, do not convey information about the value of the corresponding actions: “the quantities learned…—tendencies to perform one action over another—cannot be read out as action values” (p. 197). Instead, they are more like direct recommendations for how to (probabilistically) act.

For example, suppose a policy gradient algorithm is used to learn action weights for the children's game rock/papers/scissors, and eventually learns an optimal policy: 1/3 weight assigned to each action. These action weights do not represent the value of the respective actions. Rather, they are recommendations that one should perform each action 1/3 of the time. Hayden and Niv thus conclude that policy gradient algorithms present a counterexample for the common currency view that says that decisions for how to act are based on value, saying these algorithms “learn policies directly, that is, without calculating option values” (p. 197).

### 4.2 Why “Value-Free” RL algorithms still rely on a common currency of value

There are a pair of problems, however, for Hayden and Niv's analysis. First, notice that policy gradient algorithms are designed to acquire an *optimal* policy. Optimality in the RL framework means that an agent acting on the action weights specified in the policy achieves *maximum expected value* (relative to the case in which actions are selected on the basis of alternative action weight assignments, up to the limit of ties). So, in the rock/paper/scissors example, the optimal policy that is learned effectively conveys that doing each action 1/3 of the time will maximize expected value. It follows that policy gradient methods clearly *do* convey information about value: They convey that performing certain actions, the ones specified in the learned optimal policy, maximizes expected value.

But there is an additional deeper issue. Earlier it was noted that policy gradient algorithms work by making incremental nudges to action weights. These nudges are based on observations of subsequent cumulative rewards over a training episode. A key point that is often overlooked is that these cumulative reward signals constitute a scalar medium of value, and they play all the critical functional roles that value is supposed to play.

To see this, notice that these cumulative reward signals are used to determine the appropriate size and direction of the nudges to the action weights during each learning episode: action weight settings that yield higher cumulative reward during an episode are nudged higher, and actions weight settings that yield lower cumulative reward during an episode are nudged lower. These cumulative reward signals thus convey fine-grained magnitude-type information about the expected goodness or badness of the current action weight settings. Additionally, they allow for comparison of the goodness or badness of different action weight settings across different episodes. They additionally allow comparison of action weight settings that yield rewards from dissimilar sources (e.g., reward arising from nourishment vs. safety). In short, these cumulative reward signals quantify the scalar goodness of action settings and allow comparisons among them, precisely the roles played by a common currency of value.

Importantly, in policy gradient methods, the common currency of value operates during the *learning* phase, allowing for comparison of action weight settings in terms of value attainment and modification of these settings at each training episode. What is eventually supplied to decision-making systems is an optimal policy, i.e., the action weight settings that maximize expected value (up to the limit of ties).

The policy gradient approach contrasts with the temporal difference algorithm, which supplies magnitude-type value information at the time of decision, thus enabling comparison of the options and selection of the one with highest value. Both approaches use scalar value to compare options, but they do so at different phases. Critically, in many realistic environments, after sufficient learning, both algorithms reach the same conclusions about which are the expected value maximizing actions (Sutton et al., 1999; Agarwal et al., 2020).

A key advantage of the policy gradient approach is that it obviates the need at the time of decision for comparisons of the scalar value of the options, increasing the speed with which responses can be generated. Thus, policy gradient methods are frequently deployed for problems in which speed is at a premium, such as control of routinized movement. But the fact that magnitude-type information is not compared at the time of decision should not be taken to show that policy gradient methods eschew value altogether.

Analogous points apply to actor-critic methods (Joel et al., 2002), which can be thought of as intermediate between pure policy gradient methods and value-based temporal difference methods. In the actor–critic approach, the actor selects an action from the current policy. The critic computes a temporal difference error (or related error signal) using the observed reward and the state value estimates, and that error is used to update the policy (and also to update the value function). Actor-critic methods thus use a common currency of value, but like policy gradient methods, they use it exclusively at the learning phase.

A more general conclusion is that while some authors have spoken loosely that some RL algorithms use value information while other algorithms do not (Sutton and Barto, 1998; Sutton et al., 1999), this way of putting things is not exactly correct. *All* RL algorithms use a common currency of value to make comparisons across the options and guide decisions about how to act. What these authors likely meant is that many RL algorithms, such as the temporal difference algorithm, supply magnitude-type value information for use by decision processes during the decision-phase, but other RL algorithms, such policy gradient and actor-critic methods, leverage a common currency of value exclusively in the learning phase.

Policy gradient RL algorithms and other “value-free” methods thus do not present a problem for the common currency view. Instead, the situation is somewhat the opposite. It was noted earlier that RL algorithms of various kinds are widely observed across multiple psychological domains, including attention allocation, habitual action, working memory, and executive control. Furthermore, according to the arguments of this section, all RL algorithms use a common currency of value, some in the decision phase and others, like policy gradient and actor-critic, exclusively in the learning phase. These two observations jointly provide strong evidence *for* the common currency view.

## 5 The common currency model and competition among dissimilar psychological systems

People make decisions that trade off an astonishing diversity of goods, including food, money, safety, social gains, morally-valenced outcomes, and many others. A question arises as to how choices are made involving such highly disparate items.

The common currency view provides an attractive general answer: Disparate alternatives are compared in a common medium of value, and the alternative with the highest value is selected. But there are two importantly different levels at which a common currency can operate.

Many of the examples discussed thus far in Parts 2 and 3, including examples drawn from the RL framework, neuroimaging, and sequential sampling computational models such as decision field theory, assume what we can call an *options-level* common currency. In this approach, value attaches directly to the options themselves, i.e., the goods to be consumed, outcomes to be brought about, actions to be executed, and so forth.

While value is certainly widely used as an options-level common currency across many domains, there are principled reasons to expect limitations on this approach. There is widespread agreement that there are multiple systems in the human brain that contribute to choice and action guidance (Sanfey and Chang, 2008; van der Meer et al., 2012; Frank et al., 2009; Daw and O'Doherty, 2014). Examples include model-based RL systems, model-free RL systems, actor-critic-type systems, Pavlovian systems, and stimulus-response learning systems, among others.

The presence of multiple action guidance systems in the human brain raises the question of what happens if more than one system is concurrently engaged, and they produce divergent outputs. This question becomes especially pressing when the systems utilize very different algorithms, and their outputs are in very different formats.

Consider an example, similar to one suggested by (Hayden and Niv 2021, p. 198), in which there is competition between a model-based system and policy gradient-based system. The model-based system uses a scalar value-based RL algorithm, and it says that the expected value of option ***A*** is 12 units and option ***B*** is 4 units. The policy gradient algorithm uses a gradient-based method to learn an optimal policy representation, which presents option ***B*** as expected value maximizing. These systems operate by very different computational principles and supply different kinds of value information at the time of decision. The model-based method supplies magnitude-type expected value information for each option. The policy gradient method supplies recommended actions (or probabilistic action combinations) that are estimated to maximize expected value. Because of these differences, the outputs from the respective systems appear to be non-comparable. There does not appear to be a common currency that bridges the systems in which the options can be compared, and the one with the highest value selected. Noncomparability of this sort presents a potentially serious problem for the common currency view, which is committed to the idea that value serves as a shared medium for comparing dissimilar alternatives.

One way to address this challenge is to consider two different levels at which valuation can operate, options-level and meta-level, which yield two correspondingly different ways of executing a common currency of value. In options-level valuation, which is the type of valuation that we have been considering thus far, value is assigned directly to the bearers of value, for example goods to be consumed, outcomes to be brought about, or actions to be executed, and so forth.

In meta-level valuation, value is assigned to competing systems, not as direct bearers of value themselves, but rather as *recommendation sources*. More specifically, the agent assembles information about the expected value of relying on the recommendations of the respective systems, using indicators such as their observed prior accuracy, speed in delivering recommendations, cognitive demands, and so forth. This information is aggregated and the system with overall higher expected value as a recommendation source is selected, and its recommendation is executed. In short, instead of asking a first-order question of which option is better, the agent asks a second-order question of which system is it better to follow.

There is considerable evidence for meta-level valuation in human decision-making, which will be reviewed shortly. But here is a key point, which appears to have been underappreciated: This type of valuation allows for an elegant solution for the noncomparability problem.

Recall the earlier example of competition between a model-based RL system and a policy gradient-based RL system. Because the respective systems operate with different computational principles and supply value information in different formats, it seemed the systems yield outputs that are fundamentally non-comparable, which appears to be a violation of the common currency of value picture. However, the presence of this kind of first-order noncomparability only hampers using value to answer the first-order question of which option is better. It is no bar to asking the second-order question of which system is it better to follow. The agent can answer this higher-level question leveraging indicators such as the respective systems' prior accuracy in recommending value maximizing actions, speed in delivering recommendations, cognitive demands in accessing these recommendations, etc.

In short, even in the absence of a first-order common currency, value can still serve as a higher-order common currency allowing adjudication among competing systems in a common medium of value. Moreover, there is in fact extensive evidence that meta-level valuation is utilized in multiple domains of human cognition.

A number of researchers have investigated competition between model-based and model-free reinforcement learning, identifying a set of factors that tend to promote use of each of the respective systems. These include uncertainty in the estimates produced by the respective systems and resource demands associated with the model-based system (Daw et al., 2005; Keramati et al., 2011; Lee et al., 2014; Patzelt et al., 2019). A more recent model showed that these factors do not influence intersystem competition directly, but they instead are aggregated as a part of an overall cost/benefit calculation (Kool et al., 2017a). According to this account, model-based control is favored when its benefits in terms of accuracy outweigh its costs in terms of slower speed and opportunity cost in consuming limited cognitive resources.

A similar cost/benefit model has been put forward for explaining competition between controlled and automatic processing in the *expected value of control* (EVC) framework from Shenhav, Cohen, and colleagues (Shenhav et al., 2013, 2016; Kool et al., 2017b; Lieder et al., 2018). In this model, controlled processing is favored by cues indicating higher expected value for exercising control, which include errors on previous trials, surprising outcomes, the presence of response conflict, incentives for accurate performance, and the previous efficacy of control in yielding valued outcomes.

Additionally, another place where we see meta-level valuation is in the strategy selection phase of heuristic decision-making, which will be discussed at length in the following section.

Importantly, a number of the studies cited above explicitly tested metavaluational models based on scalar expected value against alternative simpler models that rely on specific cues or fixed heuristics. For example, Kool, Gershman, and Cushman (Kool et al., 2017a, 2016) found model-based control is favored with higher incentives for accurate performance, but not in settings in which model-based control fails to yield better accuracy. This result is consistent with an expected value model but not consistent with a simple “incentive heuristic” that increases model-based control when rewards are higher for correct responding. Similarly, Leider and colleagues tested an EVC model in which EVC is learned through an RL algorithm against simpler models that rely on associative learning or a “Win-Stay-Lose-Go” heuristic and found support for RL-based EVC model across four different experimental tests (Lieder et al., 2018). A key theme is these studies, and additional studies discussed in the following section, is that people flexibly shift which systems or strategies they utilize across contexts in ways that are better captured by scalar expected value models compared to simpler alternatives.

Finally, theorists have distinguished several different pathways by which metavaluation might operate to resolve intersystem competition (Evans, 2007; Sloman, 2014). In the “independent-competitive” view, each system produces a recommendation and metavaluation resolves which one to enact. In a “preemptive” model, metavaluation resolves which system to utilize, and that system is in turn engaged and produces a recommendation. In a “parallel-interactive” approach (Sloman, 2014), both systems activate in response to the problem context, and whichever reaches a recommendation first that is not overridden by a metavaluational quality control mechanism determines the behavior. Further empirical work is needed to distinguish which type of metavaluational approach is operative in specific domains.

In sum, there are at least two different levels at which a common currency can operate, and both are widely used in human decision-making. At the first level, dissimilar options (i.e., entities that are the direct bearers of value) compete in an options-level common currency of value to resolve the question of which option is best. At the second level, dissimilar systems compete in a meta-level common currency of value to resolve the question of which system is it best to follow. In both cases, the same underlying strategy is deployed: a common medium of value is used to resolve competition among dissimilar alternatives.

## 6 Heuristics for complex decisions: Are decision shortcuts counterexamples to the common currency view?

### 6.1 Heuristic decision-making

People often need to make choices among a large number of options that differ across multiple attributes. Canonical normative models recommend a comprehensive aggregation strategy: Value information is aggregated across the attributes of each option and this information is aggregated across the full set of options (Raiffa, 2006)—a computationally demanding endeavor. There is a sizable literature in judgment and decision-making research documenting that people often instead use heuristics, or decision shortcuts, instead of comprehensive aggregation (Tversky and Kahneman, 1974; Gigerenzer and Todd, 1999; Gigerenzer and Gaissmaier, 2011; Gilovich et al., 2002; Kahneman, 2003).

For example, in the *priority* heuristic (Brandstätter et al., 2006), decision-makers evaluate options by comparing them sequentially along attributes ordered by importance. If the difference on the first attribute exceeds a threshold, the option favored on that attribute is chosen; otherwise, the decision maker moves to the next attribute in the order, and the process repeats.

In the *elimination by aspects* heuristic (Tversky, 1972), the decision-maker identifies the most critical feature that an option should have. The option set is then culled of any options that lack this feature. If multiple options remain, the next most critical feature is identified, and another round of culling ensues. The process is repeated until only one option is left, which is the option that is selected.

### 6.2 Heuristics and the common currency view

The use of these sorts of heuristics might be seen to clash with the common currency view. This view, inspired by economic theory, says agents make choices on the basis of the value of their options, selecting the option with highest value. But an agent using heuristics ostensibly seems to be doing something quite different; they do not seem to be calculating value at all.

Hayden and Niv take up this line of critique (Hayden and Niv, 2021). They argue that whereas common currency models in the neuroeconomics framework take value to be essential for choice, heuristic approaches solve the problem of how to choose “without the need for computation of values.” They add, “Importantly for our argument, the success of heuristic approaches demonstrates that value calculations are not essential for neuroeconomic theories of choice. In particular, heuristic theories can solve many problems for which value is proposed to be needed,” including for “comparison of multi-dimensional goods and bundles, and for choice across dissimilar goods.”

Heuristics are indeed widespread in decision-making. What is up for debate, however, is the further claim, captured in the quote from Hayden and Niv, that heuristic decision-making bypasses value altogether. In what follows, it is argued that the overall picture is more nuanced, and value does enter into the overall process of heuristic decision-making.

### 6.3 Heuristic decision-making as a two-step process

The discussion by Hayden and Niv focuses on the role of value in executing a heuristic. But there is a critical prior question that goes largely unaddressed in their discussion. How does a decision-maker select a heuristic to use in the first place? Consider an agent presented with a complex decision problem involving multiple options. They could undertake an exhaustive multiattribute aggregation approach. Or they could take a heuristic approach, and there are a number of importantly different heuristic strategies available. How do agents select among all these available strategies?

This question has been the focus of decades of research in the judgment and decision-making field, and a pair of key observations are well established. First, people use a wide variety of decision-making strategies across different problem contexts (Gigerenzer and Todd, 1999; Beach and Mitchell, 1978; Payne et al., 1988). For example, sometimes they focus on important dimensions, other times they make a series of pairwise relative value comparisons, and still other times they opt for exhaustive calculation.

Second, people tend to employ strategies that are well-suited to the specific problem context at hand (Payne et al., 1988; Payne, 1993). For example, in “non-compensatory” environments, in which looking at a small set of attributes is sufficient for optimal choice, people tend to employ heuristic strategies that home in on a small set of difference-making attributes. But in “compensatory” environments, in which a large number of attributes must be considered for optimal choice, people switch to strategies that more closely resemble exhaustive multi-attribute aggregation.

These observations raise the question of how exactly people know which strategy to use for which problem context. Building on prior work (Shrager and Siegler, 1998; Erev and Barron, 2005; Rieskamp and Otto, 2006), Lieder and Griffiths (Lieder and Griffiths, 2017) propose a “metareasoning” model that understands strategy selection as a value learning problem. In their model, agents learn a predictive model that maps features of each problem type they encounter into strategies that maximize the value of computation (VOC) for that problem type. VOC in turn reflects a balance between the benefits of using a strategy, assumed to be primarily accuracy in correctly identifying the value maximizing action, and the costs, assumed to be primarily time spent executing the strategy.

The agent learns the predictive model for VOC through an RL-type iterative value learning algorithm. In this model, after employing a heuristic strategy, the agent tracks the time and effort costs in using the heuristic and also observes the consequences that ensue and updates the expected value of using the strategy in the respective context. Critically, Leider and Griffiths tested four key predictions of their RL-based metareasoning model for heuristic strategy selection across four different learning scenarios while concurrently assessing its performance against three influential alternative models that rely on simpler strategy selection methods such as associative learning. Results from their study provide support for the metareasoning model, and for its central claim that the choice to employ a heuristic strategy is itself a decision based on scaler values, which are learned through iterative RL mechanisms.

The second phase of heuristic decision-making is the heuristic execution phase. After a heuristic is selected, the agent uses the heuristic to collect decision relevant information and make a choice. For example, in the priority heuristic, the agent examines the most important dimension on which the options differ. This heuristic does rely on value in a limited way: the heuristic assumes the agent has access to scalar values for the individual attributes of options (Vlaev et al., 2011), which is needed for assessing whether one option is sufficiently better than the others on a specific dimension. Nonetheless, attribute-level scalar values are not aggregated together and overall scalar values for the options are not calculated. This is a hallmark feature of the priority heuristic and virtually all other heuristic strategies (Vlaev et al., 2011; Gigerenzer and Gaissmaier, 2011).

Importantly, however, many studies document that agents using heuristic strategies reliably select the overall best action in terms of expected value. For example, studies of the priority heuristic and other “fast and frugal” heuristics show that they often perform nearly as well, or in some cases better than, much more complex strategies in identifying optimal options (Gigerenzer et al., 2002; Hogarth and Karelaia, 2005; Katsikopoulos and Martignon, 2006; Brighton and Gigerenzer, 2012; Hogarth and Karelaia, 2007). The explanation for the effectiveness of heuristic strategies is complex and multifactorial, but a critical factor involves the adaptive heuristic selection phase discussed above (Shrager and Siegler, 1998; Erev and Barron, 2005; Rieskamp and Otto, 2006; Lieder and Griffiths, 2017): Agents learn over time which heuristics are best for which problem type, allowing them to select a heuristic that is likely to be effective for the problem at hand, which in turn allows them to select options faster and with less effort without having to sacrifice much in terms of accuracy.

### 6.4 The role of value in the overall two-step heuristic decision-making process

An important lesson from the preceding discussion of heuristic decision-making is that maximizing value does not always require exhaustive calculation of value. Instead, it is often possible to learn, via value-based learning, a shortcut decision strategy well-suited to the current context that reliably enough identifies the value maximizing action with considerably lower costs in terms of time and effort. The result is a two-step decision-making process in which the agent uses value to select among heuristics and then applies the selected heuristic to bypass value in selecting among the options.

When we think of heuristics in terms of this overall two-step process, a middle ground comes into view in which both critics and defenders of the common currency view get something important right. In employing a heuristic strategy, the agent does not choose among the options *directly* on the basis of value. This is the part that critics get right. Yet value is nonetheless importantly *implicated* in the overall two-step process of heuristic decision-making, in explaining why the agent chose to employ a heuristic, which specific heuristic is employed, and why the option selected on the basis of the heuristic is, often enough, the best in terms of expected value.

## 7 Context effects and preference reversals: Can the common currency view accommodate “irrational” choice?

The common currency model says that people select among alternatives on the basis of a common currency of value, selecting the alternative with highest value. Some theorists suggest that models of this type, which are inspired by economic theory, cannot accommodate observations of “irrational” decision-making such as context effects and preference reversals (Walasek and Brown, 2023; Vlaev, 2018; Chater and Vlaev, 2011; Tversky and Simonson, 1993).

For example, consider a decision problem that produces the so-called “similarity effect” (Huber et al., 1982). Suppose job candidate ***A*** is strong on personality and weak on work ethic while ***B*** is the reverse, and people generally prefer each roughly equally. Adding a third job candidate ***S*** who is very similar to ***A*** to the option set (slightly better than ***A*** on personality, slightly worse than ***A*** on work ethic) tends to substantially decrease the proportion of choices for ***A*** (Huber et al., 1982), while leaving the proportion choosing **B** mostly unchanged. That is, adding **S** to the context changes the proportion favoring **B** over **A**.

Observations such as the similarity effect and other preference reversals have led many theorists to question the idea of a common currency of value. For example, Walsek and Brown (Walasek and Brown, 2023) argue that, “Context-induced changes in attribute weightings are … hard to understand if there is a single common currency of value—because the existence of such a currency mandates a fixed trade-off between attribute values.” They add, “Such inconsistencies are typically taken as problematic, because utility functions cannot be easily constructed to accommodate systematic preference reversals.”

Walsek and Brown's critique, and related critiques by other theorists (Lichtenstein and Slovic, 2006; Hayden and Niv, 2021), appear to be actually directed at a different target. Their target appears to be *axiomatic* common currency models such as standard expected utility (EU) models of the kind associated with (Von Neumann and Morgenstern 1947) and Savage (1972), among others. But the common currency model at work in neuroeconomics and other areas of cognitive science, and which is the focus of this article, is a *mechanistic* common currency model.

Standard EU theory treats value as a means to parsimoniously capture patterns of preferences over options. The theory starts with an agent who is observed to display a certain pattern of preferences over pairwise options. Next, as long as these preferences satisfy a set of axiomatic consistency conditions specified in the theory, then a utility function is constructed that succinctly reflects this overall preference structure. The theory is thus *preference-reflecting* and is agnostic about the mechanisms that generate the preferences; the agent is said to behave “as if” they are maximizing expected utility. Context effects are a decisive problem for standard EU theory because inconsistent preference patterns, such as the similarity effect described above, violate these axiomatic consistency conditions. Such preferences cannot be captured by a utility function, just as the critics allege.

But the common currency model used in neuroeconomics and other areas of cognitive science is not axiomatic and preference-reflecting, it is mechanistic and preference-generating. In a mechanistic common currency model, the agent must assemble information about the options in order to locate the options on a single dimension of value. This value dimension serves as mechanism by which the goodness or badness of the options can be tabulated and compared, eventuating in observed choice behavior (i.e., observed preferences).

Critically, the information assembly process utilized to locate the options on this dimension is multi-step, unfolds over time, and relies on multiple interacting factors such as attention, memory retrieval, pairwise comparisons, noisy evidence accumulation, probability estimation, and so forth. Notably, all of these factors can influence the ongoing assembly of information in context sensitive ways, which in turn can influence subsequent choices and produce context effects.

Multi-attribute decision field theory (MDFT) provides a computationally precise illustration of this phenomenon (Roe et al., 2001). These points, however, are quite general and apply generally to other sequential sampling-type models (Busemeyer et al., 2019; Trueblood et al., 2014; Howes et al., 2016) as well as other models of complex decision-making such as heuristic decision-making (see Part 4).

In the MDFT model, the agent focuses, via a stochastic attention process, on one of the attributes of the options and compares each pair of options along this attribute dimension. In the job candidate example discussed earlier, if the attribute currently in focus were work ethic, ***B*** would strongly beat ***A*** and ***S***, while if the attribute in focus were personality, ***A*** and ***S*** would strongly beat ***B***. As attention moves from attribute to attribute, value information arising from these pairwise comparisons accumulates for each option over time under conditions of noise. When a prespecified confidence threshold is reached, the agent selects the associated option.

Importantly, the MDFT model provides a natural explanation for the similarity effect in terms of division of attentional advantages (Roe et al., 2001). When attention focuses more on the work ethic dimension, ***B*** uniquely gains an advantage, but when attention focuses more on the personality dimension, *both* ***A*** and ***S*** gain an advantage, and once we add noise into the picture, the “wins” are roughly split between them. Thus, the addition of ***S*** to the option set tends to leave the proportion of choices of ***B*** unchanged while choices for ***A*** are roughly halved—precisely the contextual effect that is observed in experimental settings.

For our purposes, the key point is that models like MDFT explain how context effects can arise in terms of the assembly of information in order to locate options on a mechanistic common currency of value. The information assembly process often relies on factors such as stochastic attention, pairwise value comparisons, noisy evidence accumulation, etc., which are susceptible to contextual influences and can thus produce context effects and preference reversals.

Overall, then, the critics are right that context effects are not compatible with one kind of common currency model, standard expected utility theory, an axiomatic, preference-reflecting theory that requires that choices obey certain consistency conditions in order to be seen as maximizing utility. But the common currency model at work in cognitive science is not the axiomatic, preference-reflecting expected utility theory model, it is a mechanistic, preference-generating common currency model. As the detailed example using MDFT illustrates, models of this type that utilize a mechanistic common currency of value are compatible with context effects and preference reversals, and indeed provide an explanation for how they arise.

## 8 Conclusion: the overall case for the common currency view

The common currency view is a core thesis in neuroeconomics and other areas of cognitive science. It finds support from behavioral, chronometric, computational, and neural methods; and it has been applied across diverse domains of cognition. The evidence base for the view has been documented in detail in previous reviews (Montague and Berns, 2002; Glimcher and Rustichini, 2004; Sanfey et al., 2006; Glimcher and Fehr, 2013), and key threads were summarized in section 2 of this article.

The main focus of this article has been on four major criticisms of the common currency view that have emerged in the recent literature. The essence of the response was that these criticisms are focused on a relatively simple kind of common currency model in which the agent always calculates the scalar value of their options at the time of decision—a model that admittedly encounters significant problems and is rightfully criticized. Over the course of this article, a more sophisticated version of the common currency model was elaborated and defended.

Three key distinctions were made in how a common currency of value can be implemented. First, a distinction was made between common currencies that operate at the decision phase vs. at the learning phase. This distinction helped address that the objection that certain “value-free” RL algorithms bypass a common currency of value; they bypass it only at the decision phase, but they critically rely on scalar value signals during the learning phase to establish which are the expected value maximizing actions. Second, a distinction was made between two levels of valuation, options-level and meta-level. Meta-level valuation helps address cases in which dissimilar psychological systems compete and also sheds light on the role of value in heuristic decision-making.

A distinction was also made between mechanistic, preference-generating common currency models utilized in cognitive science and axiomatic, preference-reflecting common currency models utilized in standard EU theory. This distinction is critical to address the claim that choice anomalies such as context effects and preference reversals are incompatible with the common currency view. It was argued that they are only incompatible with axiomatic common currency models, and mechanistic common currency models, such as MDFT, actually predict and explain these effects. Overall, the more nuanced common currency view defended in this article is more psychologically realistic, and it offers ways to address a number of important recent criticisms directed at the common currency view.

Many of the of the arguments of this article rely on key empirical premises, and it should be acknowledged that the state of the overall empirical record remains contested and is evolving. For example, in sections 2 and 3, it was argued that the common currency view is supported by evidence of the presence of RL mechanisms in numerous domains, including attention allocation, working memory, action selection, and routinized movement. However, some theorists offer alternative non-RL explanations for selection among options in some of these domains (Robinson and Berridge, 2013; Berridge, 2023; Miller et al., 2019). There are in addition very general alternatives to the overall RL paradigm. One of the best developed is the predictive processing framework, which is claimed by advocates to explain selection among options without the need for RL-style value representations (Friston, 2010; Friston et al., 2017, 2009; Sajid et al., 2021). However, other work suggests that to fully capture empirical datapoints about decision, predictive processing still requires a fundamental value signal that cannot be reduced to prediction error or uncertainty reduction. Additionally, whether “value-free” predictive processing in fact dispenses with value representations, or instead covertly relies on them, is itself contested (Smith et al., 2022).

Other empirical premises concern the role of scalar value in intersystem arbitration and heuristic strategy selection. Evidence for scalar value-based metavaluation mechanisms was reviewed in sections 4 and 5. Moreover, several studies were reviewed that performed head-to-head comparisons of models based on scalar value and alternatives that rely on simpler methods such as associative learning or fixed cues (Kool et al., 2017a, 2016; Lieder et al., 2018; Lieder and Griffiths, 2017). While such studies are important, the overall evidence base is certainly not decisive.

The fact that the common currency model relies on the preceding empirical premises should go some way in addressing a frequently heard complaint: that the view is unfalsifiable. Against this charge, the common currency view advanced in this article puts forward specific mechanistic hypotheses about how value is learned (e.g., RL mechanisms), how options are compared and chosen (e.g., MDFT), how intersystem arbitration unfolds (e.g., cost/benefit models), how heuristic strategies are selected (e.g., metareasoning models), and so forth. Evidence for these hypotheses was reviewed in sections 3 through 6 of the paper, but, as was noted above, these hypotheses remain contested. Ultimately, if sufficient evidence is eventually amassed to overturn all, or most of, the preceding mechanistic hypotheses that the common currency view relies on, then the common currency view will be shown to be false.

Finally, the approach of this article has been to view the debate about the common currency view as a series of potentially separable debates about whether, and to what extent, the view applies to individual areas or domains of human psychology. If the arguments of this article are correct, then the scope of the common currency view is indeed quite broad.

For example, the common currency view applies to choices in which value information is supplied by diverse kinds of RL algorithms, including policy gradient and actor-critic algorithms which are sometimes described as “value free” (and evidence was also reviewed in section 2 that RL algorithms are in fact operative across a sizable number of psychological domains). The view applies to cases in which disparate options are directly traded off, but, as argued in section 4, it also applies to more complex situations in which dissimilar psychological systems compete. The model additionally extends to “irrational” choices involving contextual effects and preference reversals—these effects are well explained by models such as MDFT that utilize a mechanistic common currency of value. An important exception, however, was the case of heuristic decision-making, where it was conceded that an agent employing a heuristic strategy does not *directly* select among options on the basis of value. Yet, it was argued that full picture is more nuanced, and value is nonetheless deeply implicated in the overall two-step process of heuristic strategy selection and strategy execution.

Overall, the common currency view appears to have substantial scope across a number of important psychological domains. But the view admits of important exceptions, and its precise scope is the subject of ongoing debate and awaits further clarification.

## Data Availability

The original contributions presented in the study are included in the article/supplementary material, further inquiries can be directed to the corresponding author.
